# (1*S*,3*S*,8*R*,9*S*,10*R*)-9,10-Ep­oxy-3,7,7,10-tetra­methyl­tri­cyclo­[6.4.0.0^1,3^]dodeca­ne

**DOI:** 10.1107/S1600536814006230

**Published:** 2014-03-26

**Authors:** Abdoullah Bimoussa, Aziz Auhmani, My Youssef Ait Itto, Jean-Claude Daran, Abdelwahed Auhmani

**Affiliations:** a Laboratoire de Synthése Organique et Physico-Chimie Moléculaire, Département de Chimie, Faculté des Sciences Semlalia, BP 2390 Marrakech 40000, Morocco; b Laboratoire de Chimie de Coordination, 205 route de Narbonne, 31077 Toulouse Cedex 04, France

## Abstract

The title compound, C_16_H_26_O, was synthesized by treating (1*S*,3*S*,8*R*)-3,7,7,10-tetra­methyl­tri­cyclo­[6.4.0.0^1,3^]dodec-9-ene with meta­chloro­perbenzoic acid. The mol­ecule is built up from two fused six- and seven-membered rings. The six-membered ring has a half-chair conformation, whereas the seven-membered ring displays a boat conformation. In the crystal, there are no significant intermolecular interactions present.

## Related literature   

For the use of ep­oxy­des in organic synthesis, see: Mori (1989 ▶); Paddon-Jones *et al.* (1997 ▶); Taylor *et al.* (1991 ▶). For their biological activity, see: Kupchan *et al.* (1989 ▶); Trost *et al.* (1983 ▶); Vollhardt & Schore (1996 ▶); Yang (2004 ▶). For structural discussion, see: Cremer & Pople (1975 ▶); Flack (1983 ▶); Flack & Bernardinelli (2000 ▶); Spek (2009 ▶); Boessenkool & Boyens (1980 ▶); Benharref *et al.* (2010 ▶). For the synthesis, see: Auhmani *et al.* (2001 ▶).
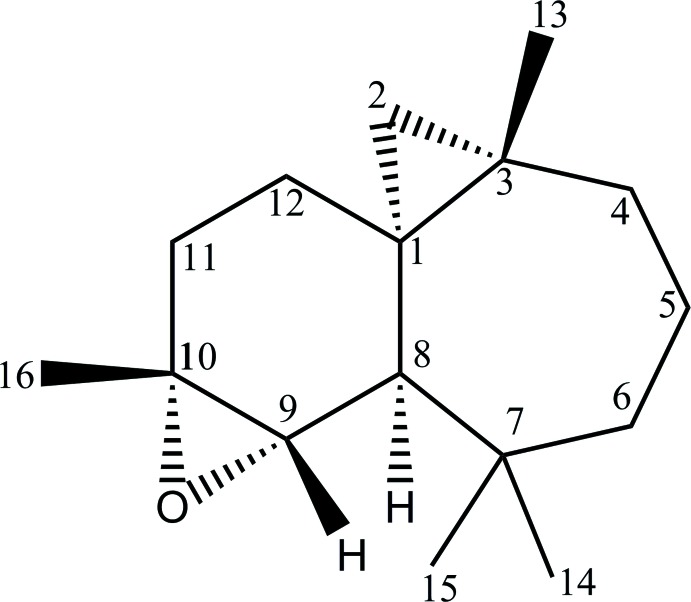


## Experimental   

### 

#### Crystal data  

C_16_H_26_O
*M* *_r_* = 234.37
Monoclinic, 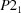

*a* = 10.5563 (10) Å
*b* = 5.7548 (5) Å
*c* = 11.7096 (13) Å
β = 92.777 (8)°
*V* = 710.52 (12) Å^3^
*Z* = 2
Mo *K*α radiation
μ = 0.07 mm^−1^
*T* = 180 K
0.31 × 0.31 × 0.25 mm

#### Data collection  

Agilent Xcalibur Eos Gemini ultra diffractometer
Absorption correction: multi-scan (*CrysAlis PRO*; Agilent, 2012 ▶) *T*_min_ = 0.767, *T*_max_ = 1.0
8241 measured reflections
2899 independent reflections
2150 reflections with *I* > 2σ(*I*)
*R*_int_ = 0.055

#### Refinement  

*R*[*F* ^2^ > 2σ(*F* ^2^)] = 0.054
*wR*(*F* ^2^) = 0.105
*S* = 1.05
2899 reflections
158 parameters
1 restraint
H-atom parameters constrained
Δρ_max_ = 0.14 e Å^−3^
Δρ_min_ = −0.21 e Å^−3^

### 

Data collection: *CrysAlis PRO* (Agilent, 2012 ▶); cell refinement: *CrysAlis PRO*; data reduction: *CrysAlis PRO*; program(s) used to solve structure: *SIR97* (Altomare *et al.*, 1999 ▶); program(s) used to refine structure: *SHELXL2013* (Sheldrick, 2008 ▶); molecular graphics: *ORTEPIII* (Burnett & Johnson, 1996 ▶) and *ORTEP-3 for Windows* (Farrugia, 2012 ▶); software used to prepare material for publication: *SHELXL2013*.

## Supplementary Material

Crystal structure: contains datablock(s) I, New_Global_Publ_Block. DOI: 10.1107/S1600536814006230/xu5778sup1.cif

Structure factors: contains datablock(s) I. DOI: 10.1107/S1600536814006230/xu5778Isup2.hkl

Supporting information file. DOI: 10.1107/S1600536814006230/xu5778Isup3.cml

CCDC reference: 992783

Additional supporting information:  crystallographic information; 3D view; checkCIF report

## Figures and Tables

**Fig. 1.**
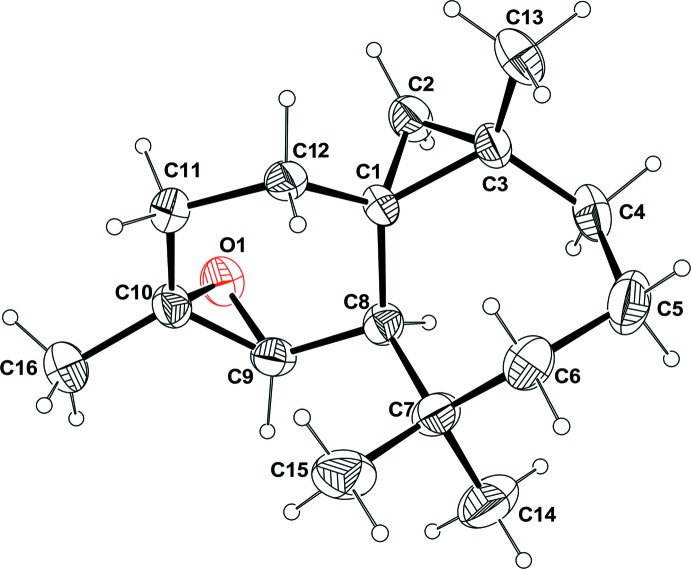
: Molecular view of the title compound with the atom labeling scheme. Ellipsoids are drawn at the 50% probability level. H atoms are represented as small spheres of arbitrary radii.

## supplementary crystallographic information

### 1. Comment

Epoxides are important synthetic intermediates that are widely used in organic synthesis. They are described in the synthesis of Antifungal products (Taylor *et al.*, 1991) and different pheromones (Mori, 1989, Paddon-Jones *et al.*, 1997). Besides, many natural products possess this functional group as an essential structural moiety for their biological activities (Yang, 2004; Vollhardt *et al.*, 1996; Trost *et al.*, 1983; Kupchan *et al.*, 1974). Because of their widespread occurrence and synthetic utility, the development of methods for the direct asymmetric synthesis of epoxides has grown significantly. In order to prepare new epoxides with natural products, we synthesized (1*S*,3S,8*R*,9S,10*R*)-9,10-epoxy-3,7,7,10-tetramethyltricyclo [6.4.0.0^1,3^]dodecane in three stages from β-himachalene. The title compound was prepared by treating (1*S*,3*S*,8*R*)-3,7,7,10-tetramethyltricyclo[6.4.0.0^1,3^]dodec-9-ene (Auhmani *et al.*, 2001) by metachloroperbenzoique acide.

The title compound is built up from two fused six and seven-membered rings (Fig. 1). The six-membered-ring has an half chair conformation with puckering parameters: Q = 0.447 (3) Å, θ= 128.5 (4)° and φ= 171.6 (6)° (Cremer & Pople, 1975), whereas the seven-membered ring displays a boat conformation with puckering amplitudes: Q2 = 1.142 (4) and Q3 =0.036 (4) (Boessenkool & Boyens, 1980). Although the absolute configuration is different, this structure is closely related to the (1*S*,3S,8*R*,9S,10*R*)-2,2-Dichloro-3,7,7,10-tetramethyl-9,10-epoxytricyclo [6.4.0.0^1,3^]dodecane (Benharref *et al.*, 2010) however the seven-membered ring displays a chair conformation.

The absolute configuration (1*S*,3*S*,8*R*,9*S*,10*R*) is deduced from the chemical pathway. The refinement of the Flack's parameter (-0.2 (10)) (Flack, 1983; Flack & Bernardinelli, 2000) as well as the Hooft's parameter ((Spek, 2009) do not allow to define reliably the absolute configuration.

### 2. Experimental

In 100 mL flask containing (0.220 g, 1.009 mmol) of (1*S*,3*S*,8*R*)-3,7,7,10-tetramethyltricyclo[6.4.0.0^1,3^]dodec-9-ene in 20 ml of dichloromethane was added a stoechiometric quantity of *m*-chloroperbenzoic acid (*m*-CPBA). The reaction mixture was stirred at room temperature for 2 h and then treated with 10% solution of sodium hydrogencarbonate. The reaction mixture was extracted with dichloromethane (3x 20 mL) and the organic layer were dried over anhydrous Na_2_SO_4_ and concentrated under reduced pressure. The crude product was purified by chromatography on silica gel (230–400 mesh) with Hexane/Ethyl acetate (97:3) as eluent to give the title compound (1*S*,3S,8*R*,9S,10*R*)-9,10-epoxy-3,7,7,10-tetramethyltricyclo [6.4.0.0^1,3^]dodecane in 72% yield. X-ray quality crystals were obtained by slow evaporation from a petroleum ether solution of the title compound.

### 3. Refinement

All H atoms were fixed geometrically and treated as riding with C—H = 0.98 Å (methyl), 0.99 Å (methylene) and 1.00 Å (methine) In the absence of significant anomalous scattering, the absolute configuration could not be reliably determined and any references to the Flack parameter were removed.

### Crystal data

**Table:**

C_16_H_26_O	*F*(000) = 260
*M**_r_* = 234.37	*D*_x_ = 1.095 Mg m^−^^3^
Monoclinic, *P*2_1_	Mo *K*α radiation, λ = 0.71073 Å
Hall symbol: P 2yb	Cell parameters from 1871 reflections
*a* = 10.5563 (10) Å	θ = 3.9–26.5°
*b* = 5.7548 (5) Å	µ = 0.07 mm^−^^1^
*c* = 11.7096 (13) Å	*T* = 180 K
β = 92.777 (8)°	Box, colourless
*V* = 710.52 (12) Å^3^	0.31 × 0.31 × 0.25 mm
*Z* = 2	

### Data collection

**Table:**

Agilent Xcalibur Eos Gemini ultra diffractometer	2899 independent reflections
Radiation source: Enhance (Mo) X-ray Source	2150 reflections with *I* > 2σ(*I*)
Graphite monochromator	*R*_int_ = 0.055
Detector resolution: 16.1978 pixels mm^-1^	θ_max_ = 26.4°, θ_min_ = 3.5°
ω scans	*h* = −13→13
Absorption correction: multi-scan (*CrysAlis PRO*; Agilent, 2012)	*k* = −7→7
*T*_min_ = 0.767, *T*_max_ = 1.0	*l* = −14→14
8241 measured reflections	

### Refinement

**Table:**

Refinement on *F*^2^	1 restraint
Least-squares matrix: full	Hydrogen site location: inferred from neighbouring sites
*R*[*F*^2^ > 2σ(*F*^2^)] = 0.054	H-atom parameters constrained
*wR*(*F*^2^) = 0.105	*w* = 1/[σ^2^(*F*_o_^2^) + (0.0267*P*)^2^] where *P* = (*F*_o_^2^ + 2*F*_c_^2^)/3
*S* = 1.05	(Δ/σ)_max_ < 0.001
2899 reflections	Δρ_max_ = 0.14 e Å^−^^3^
158 parameters	Δρ_min_ = −0.21 e Å^−^^3^

### Special details

**Table:**

Geometry. All e.s.d.'s (except the e.s.d. in the dihedral angle between two l.s. planes) are estimated using the full covariance matrix. The cell e.s.d.'s are taken into account individually in the estimation of e.s.d.'s in distances, angles and torsion angles; correlations between e.s.d.'s in cell parameters are only used when they are defined by crystal symmetry. An approximate (isotropic) treatment of cell e.s.d.'s is used for estimating e.s.d.'s involving l.s. planes.

### Fractional atomic coordinates and isotropic or equivalent isotropic displacement parameters (Å^2^)

**Table:**

	*x*	*y*	*z*	*U*_iso_*/*U*_eq_	
C1	0.2747 (3)	0.7760 (5)	0.3324 (3)	0.0241 (7)	
C2	0.2251 (3)	0.6047 (6)	0.4168 (3)	0.0334 (8)	
H2A	0.2213	0.4390	0.3941	0.040*	
H2B	0.2461	0.6322	0.4990	0.040*	
C3	0.1336 (3)	0.7699 (5)	0.3563 (3)	0.0276 (7)	
C4	0.0457 (3)	0.6751 (6)	0.2618 (3)	0.0392 (9)	
H4A	−0.0380	0.6418	0.2929	0.047*	
H4B	0.0805	0.5271	0.2338	0.047*	
C5	0.0281 (3)	0.8450 (7)	0.1615 (3)	0.0493 (11)	
H5A	−0.0017	0.7571	0.0927	0.059*	
H5B	−0.0391	0.9578	0.1791	0.059*	
C6	0.1479 (3)	0.9792 (7)	0.1341 (3)	0.0429 (9)	
H6A	0.1271	1.0768	0.0661	0.051*	
H6B	0.1680	1.0860	0.1987	0.051*	
C7	0.2684 (3)	0.8433 (6)	0.1112 (3)	0.0335 (8)	
C8	0.3116 (3)	0.6867 (5)	0.2160 (3)	0.0265 (8)	
H8	0.2675	0.5342	0.2043	0.032*	
C9	0.4518 (3)	0.6354 (5)	0.2177 (3)	0.0295 (8)	
H9	0.4857	0.5925	0.1423	0.035*	
C10	0.5427 (3)	0.7440 (6)	0.2993 (3)	0.0289 (8)	
C11	0.4987 (3)	0.9110 (5)	0.3879 (3)	0.0296 (8)	
H11A	0.5481	1.0566	0.3826	0.036*	
H11B	0.5182	0.8436	0.4645	0.036*	
C12	0.3575 (3)	0.9715 (5)	0.3781 (3)	0.0274 (7)	
H12A	0.3296	1.0162	0.4546	0.033*	
H12B	0.3454	1.1078	0.3272	0.033*	
C13	0.0746 (3)	0.9619 (6)	0.4244 (3)	0.0381 (9)	
H13A	0.0582	1.0970	0.3750	0.057*	
H13B	0.1328	1.0060	0.4885	0.057*	
H13C	−0.0054	0.9069	0.4539	0.057*	
C14	0.2449 (4)	0.6836 (8)	0.0066 (3)	0.0551 (11)	
H14A	0.1789	0.5702	0.0227	0.083*	
H14B	0.3235	0.6018	−0.0094	0.083*	
H14C	0.2174	0.7774	−0.0599	0.083*	
C15	0.3718 (3)	1.0198 (6)	0.0830 (3)	0.0460 (10)	
H15A	0.3407	1.1195	0.0198	0.069*	
H15B	0.4478	0.9367	0.0608	0.069*	
H15C	0.3928	1.1157	0.1505	0.069*	
C16	0.6798 (3)	0.7660 (7)	0.2706 (3)	0.0460 (10)	
H16A	0.7340	0.7499	0.3406	0.069*	
H16B	0.6939	0.9186	0.2363	0.069*	
H16C	0.7007	0.6438	0.2164	0.069*	
O1	0.50267 (19)	0.5041 (4)	0.31494 (19)	0.0350 (6)	

### Atomic displacement parameters (Å^2^)

**Table:**

	*U*^11^	*U*^22^	*U*^33^	*U*^12^	*U*^13^	*U*^23^
C1	0.0183 (15)	0.0264 (17)	0.0278 (18)	−0.0012 (14)	0.0043 (13)	−0.0014 (15)
C2	0.0307 (19)	0.0345 (19)	0.035 (2)	0.0008 (16)	0.0051 (15)	0.0065 (16)
C3	0.0200 (16)	0.0298 (18)	0.0331 (19)	−0.0018 (15)	0.0035 (14)	0.0032 (16)
C4	0.0207 (18)	0.044 (2)	0.053 (3)	−0.0058 (16)	0.0012 (16)	−0.0024 (19)
C5	0.032 (2)	0.063 (3)	0.051 (3)	0.006 (2)	−0.0125 (18)	−0.001 (2)
C6	0.039 (2)	0.052 (2)	0.036 (2)	0.008 (2)	−0.0060 (16)	0.012 (2)
C7	0.036 (2)	0.040 (2)	0.0245 (19)	0.0031 (17)	−0.0014 (15)	0.0003 (16)
C8	0.0259 (18)	0.0267 (18)	0.0267 (19)	−0.0016 (14)	0.0000 (14)	−0.0050 (14)
C9	0.0309 (19)	0.0325 (19)	0.0254 (19)	0.0047 (16)	0.0049 (14)	0.0018 (16)
C10	0.0229 (17)	0.0339 (19)	0.0299 (19)	0.0008 (16)	0.0008 (14)	0.0046 (16)
C11	0.0251 (17)	0.034 (2)	0.0295 (19)	−0.0038 (14)	−0.0014 (14)	−0.0024 (15)
C12	0.0281 (18)	0.0302 (17)	0.0242 (18)	−0.0042 (16)	0.0028 (13)	−0.0060 (15)
C13	0.0273 (19)	0.036 (2)	0.052 (2)	0.0061 (16)	0.0123 (16)	−0.0024 (18)
C14	0.065 (3)	0.069 (3)	0.030 (2)	0.006 (2)	−0.0101 (19)	−0.010 (2)
C15	0.055 (2)	0.046 (2)	0.037 (2)	0.002 (2)	0.0087 (17)	0.0144 (18)
C16	0.0251 (19)	0.071 (3)	0.043 (2)	0.000 (2)	0.0038 (16)	−0.001 (2)
O1	0.0302 (13)	0.0314 (12)	0.0433 (15)	0.0050 (12)	0.0011 (10)	0.0018 (12)

### Geometric parameters (Å, º)

**Table:**

C1—C12	1.507 (4)	C9—O1	1.448 (4)
C1—C2	1.508 (4)	C9—C10	1.462 (4)
C1—C8	1.525 (4)	C9—H9	1.0000
C1—C3	1.529 (4)	C10—O1	1.458 (4)
C2—C3	1.507 (4)	C10—C11	1.504 (4)
C2—H2A	0.9900	C10—C16	1.507 (4)
C2—H2B	0.9900	C11—C12	1.530 (4)
C3—C4	1.511 (5)	C11—H11A	0.9900
C3—C13	1.514 (4)	C11—H11B	0.9900
C4—C5	1.533 (5)	C12—H12A	0.9900
C4—H4A	0.9900	C12—H12B	0.9900
C4—H4B	0.9900	C13—H13A	0.9800
C5—C6	1.529 (5)	C13—H13B	0.9800
C5—H5A	0.9900	C13—H13C	0.9800
C5—H5B	0.9900	C14—H14A	0.9800
C6—C7	1.528 (4)	C14—H14B	0.9800
C6—H6A	0.9900	C14—H14C	0.9800
C6—H6B	0.9900	C15—H15A	0.9800
C7—C15	1.538 (5)	C15—H15B	0.9800
C7—C14	1.541 (5)	C15—H15C	0.9800
C7—C8	1.573 (4)	C16—H16A	0.9800
C8—C9	1.508 (4)	C16—H16B	0.9800
C8—H8	1.0000	C16—H16C	0.9800
			
C12—C1—C2	118.0 (3)	O1—C9—C8	116.1 (2)
C12—C1—C8	113.6 (2)	C10—C9—C8	122.5 (3)
C2—C1—C8	118.5 (3)	O1—C9—H9	115.5
C12—C1—C3	120.4 (2)	C10—C9—H9	115.5
C2—C1—C3	59.52 (19)	C8—C9—H9	115.5
C8—C1—C3	116.7 (3)	O1—C10—C9	59.45 (19)
C3—C2—C1	60.9 (2)	O1—C10—C11	114.7 (2)
C3—C2—H2A	117.7	C9—C10—C11	120.6 (3)
C1—C2—H2A	117.7	O1—C10—C16	113.3 (3)
C3—C2—H2B	117.7	C9—C10—C16	119.9 (3)
C1—C2—H2B	117.7	C11—C10—C16	115.6 (3)
H2A—C2—H2B	114.8	C10—C11—C12	115.2 (3)
C2—C3—C4	118.3 (3)	C10—C11—H11A	108.5
C2—C3—C13	118.9 (3)	C12—C11—H11A	108.5
C4—C3—C13	113.3 (3)	C10—C11—H11B	108.5
C2—C3—C1	59.5 (2)	C12—C11—H11B	108.5
C4—C3—C1	116.3 (3)	H11A—C11—H11B	107.5
C13—C3—C1	120.6 (3)	C1—C12—C11	113.8 (2)
C3—C4—C5	112.2 (3)	C1—C12—H12A	108.8
C3—C4—H4A	109.2	C11—C12—H12A	108.8
C5—C4—H4A	109.2	C1—C12—H12B	108.8
C3—C4—H4B	109.2	C11—C12—H12B	108.8
C5—C4—H4B	109.2	H12A—C12—H12B	107.7
H4A—C4—H4B	107.9	C3—C13—H13A	109.5
C6—C5—C4	114.3 (3)	C3—C13—H13B	109.5
C6—C5—H5A	108.7	H13A—C13—H13B	109.5
C4—C5—H5A	108.7	C3—C13—H13C	109.5
C6—C5—H5B	108.7	H13A—C13—H13C	109.5
C4—C5—H5B	108.7	H13B—C13—H13C	109.5
H5A—C5—H5B	107.6	C7—C14—H14A	109.5
C7—C6—C5	118.9 (3)	C7—C14—H14B	109.5
C7—C6—H6A	107.6	H14A—C14—H14B	109.5
C5—C6—H6A	107.6	C7—C14—H14C	109.5
C7—C6—H6B	107.6	H14A—C14—H14C	109.5
C5—C6—H6B	107.6	H14B—C14—H14C	109.5
H6A—C6—H6B	107.0	C7—C15—H15A	109.5
C6—C7—C15	107.8 (3)	C7—C15—H15B	109.5
C6—C7—C14	110.0 (3)	H15A—C15—H15B	109.5
C15—C7—C14	108.2 (3)	C7—C15—H15C	109.5
C6—C7—C8	111.6 (3)	H15A—C15—H15C	109.5
C15—C7—C8	111.3 (3)	H15B—C15—H15C	109.5
C14—C7—C8	107.9 (3)	C10—C16—H16A	109.5
C9—C8—C1	110.3 (3)	C10—C16—H16B	109.5
C9—C8—C7	111.7 (2)	H16A—C16—H16B	109.5
C1—C8—C7	115.3 (2)	C10—C16—H16C	109.5
C9—C8—H8	106.3	H16A—C16—H16C	109.5
C1—C8—H8	106.3	H16B—C16—H16C	109.5
C7—C8—H8	106.3	C9—O1—C10	60.41 (19)
O1—C9—C10	60.14 (19)		
